# TRPM6 in murine kidneys—of targets and antibodies

**DOI:** 10.1007/s00210-025-03951-0

**Published:** 2025-03-01

**Authors:** Colya N. Englisch, Coline M. Diebolt, Emilie Kirstein, Vanessa Wahl, Philipp Wartenberg, Dirk Schaudien, Anja Beckmann, Matthias W. Laschke, Gabriela Krasteva-Christ, Thomas Gudermann, Vladimir Chubanov, Ulrich Boehm, Thomas Tschernig

**Affiliations:** 1 Institute of Anatomy and Cell Biology, Saarland University, Kirrberger Strasse 100, 66421 Homburg/Saar, Germany; 2 Institute of Pharmacology, Saarland University, 66421 Homburg/Saar, Germany; 3 Fraunhofer Institute for Toxicology and Experimental Medicine, 30625 Hannover, Germany; 4 Institute for Clinical and Experimental Surgery, Saarland University, 66421 Homburg/Saar, Germany; 5 Walther-Straub Institute of Pharmacology and Toxicology, Ludwig Maximilian University Munich, Munich, Germany

**Keywords:** TRPM6, Kidney, Mouse, Antibodies, Immunohistochemistry, Immunofluorescence, Knockout

## Abstract

Magnesium is the fourth most abundant cation in the human organism. As a key-player in many enzymatic reactions, magnesium homeostasis disbalance can cause severe disorders. In the early 2000s, the transient receptor potential melastatin channel 6 (TRPM6) was identified as a critical protein in renal Mg^2+^-reabsorption in the distal convoluted tubule (DCT). As the key-interface responsible for salt/water adaptation to environmental changes, the kidney is a highly dynamic system. Therefore, renal TRPM6 expression and Mg^2+^-reabsorption might not be restricted to the DCT, as previously indicated. To address this, protein targeting is mandatory since genomic detection is insufficient to conclude on functional expression. For this purpose, we used a polyclonal TRPM6 antibody from an established manufacturer and detected immunostaining in murine proximal and distal tubules. As a matter of fact, the specificity of most commercially available TRPM6 antibodies is insufficiently validated which relies on the lack of constitutive *trpm6* knockouts. Therefore, conditional *trpm6* knockout mice were used for control experiments. Similar signals were observed in the knockout tissue when compared to wildtype using the TRPM6 antibody. Overlaps with TRPM7 epitopes or other peptides are conceivable. Thus, TRPM6 immunohistochemistry and immunofluorescence results are difficult to interpret, and the spectrum of renal TRPM6 expression is not yet elucidated.

## Introduction

Transient receptor potential (TRP) channels constitute a family of heterogeneous ion channels. Most representatives are crucially involved in tissue type-dependent physiology, including the cardiovascular, pulmonal, neurosensory, and renal systems (Yue et al. 2015; Tomilin et al. 2016; Dietrich et al. 2017; Englisch et al. 2022a, 2023). Progressively, a subclassification based on protein homologies has been established and includes the TRPM (melastatin), TRPC (canonical), TRPV (vanilloid), TRPP (polycystin), TRPA (ankyrin), TRPML (mucolipin), and TRPN (no mechanoreceptor potential C) subfamilies (Montell et al. 2002; Nilius and Owsianik 2011). Nevertheless, TRP channels share a rudimentary scaffold consisting of four subunits. Each of these subunits features six transmembrane segments (S1–S6) and contributes to the ion-pore formation with an α-helix loop between S5 and S6 (Jimenez et al. 2020). The corresponding cytosolic COOH (C)- and NH_2_ (N)-termini can carry multiple domains in different combinations or variations and thereby contribute to the high heterogenicity among TRP channels (Jimenez et al. 2020). The aforementioned domains include coiled-coils, ankyrin-like repeats, and TRP-boxes (Venkatachalam and Montell 2007). The TRP-box, for instance, serves as C-terminus-based stabilizer and linking-tool for other TRP-isoforms and specifically characterizes TRPC and TRPM channels (Jiang 2007; Birnbaumer 2009; Jimenez et al. 2020). TRPC and TRPM channels are important in renal physiology and pathophysiology (Nijenhuis et al. 2004; Voets et al. 2004; Englisch et al. 2022a, 2024a, 2022b; Diebolt et al. 2024). TRPC3, for instance, is suggested to play a critical role in calcium homeostasis (Ibeh et al. 2019; Awuah Boadi et al. 2021; Englisch et al. 2022a, 2024a; Diebolt et al. 2024; Kirstein et al. 2024b), while TRPM6 is rather crucial in magnesium homeostasis (Nijenhuis et al. 2004; Voets et al. 2004). Uniform patterns including melastatin homology regions (MHR) at the N-terminus, a coiled-coil domain, and the TRP-box at the C-terminus label, the TRPM subfamily (Jimenez et al. 2020). TRPM6 and TRPM7 further evolve to analogue heteropolimerizable bifunctional units—both proteins being additionally equipped with two C-terminus-bound enzymatic serin/threonine rich and α-kinase domains that are mandatory for, e.g., regulatory autophosphorylation (Riazanova et al. 2001; Clark et al. 2008; Fujiwara and Minor 2008; Jimenez et al. 2020). TRPM6 and TRPM7 are highly permeable to Ca^2+^ and Mg^2+^ and indeed critical in calcium and magnesium homeostasis, as indicated above (Li et al. 2006; Chubanov and Gudermann 2014; Chubanov et al. 2018). Channel-gating is regulated by a multitude of activating (TRPM6: e.g., phosphatidylinositol-4,5-bisphosphate [PIP_2_] and phospholipase C signaling [PLC], epidermal growth factor/extracellular signal-regulated kinase/activator protein-1 [EGF/ERK/AP-1] signaling, etc.) and inhibiting (TRPM6: e.g., P_2_ purinoreceptor 4 [P_2_X_4_R] activity, metformin, mutations, and polymorphisms) mechanisms (Jimenez et al. 2020). In contrast to TRPM7, TRPM6 expression is predominantly restricted to the kidneys and the gut (Chubanov et al. 2004; Fonfria et al. 2006; Groenestege et al. 2006; Schlingmann et al. 2007). Here, mutations in the *trpm6* gene have been identified to cause hypomagnesemia with secondary hypocalcemia (HSH) (Schlingmann et al. 2002). Little later, TRPM6 was described as the Mg^2+^-influx channel responsible for apical magnesium reabsorption in the distal convoluted tubule (DCT) of the kidney (Voets et al. 2004). The role of TRPM6 in renal magnesium reabsorption was further supported by states of combined hypomagnesemia and hypermagnesuria after drug-triggered TRPM6 downregulation (Nijenhuis et al. 2004).

Magnesium is the fourth most abundant cation in vertebrates and more precisely the second most abundant intracellular cation after potassium (Jahnen-Dechent and Ketteler 2012). Mg^2+^ is a key component of numerous enzymatic reactions and therefore essential in organism’s physiology. Impairment of transmembrane Mg^2+^-channeling enhances severe systemic homeostasis disbalance (Jahnen-Dechent and Ketteler 2012; DiNicolantonio et al. 2018; Jimenez et al. 2020). HSH, for instance,—characterized by reduced Mg^2+^-uptake in the gut and insufficient “recycling” Mg^2+^-reabsorption in the renal tubules—is accompanied by neuromuscular disorders (Schlingmann et al. 2002; Jimenez et al. 2020).

The kidney is responsible for salt/water adaptation to environmental changes. If it was not such a highly dynamic system, life on land would be impossible. To that end, it seems natural that the expression of tubular channels and transporters is subject to extensive variations depending on inner and outer circumstances. This is the reason why renal TRPM6 expression and Mg^2+^-reabsorption might not be restricted to the DCT, as, however, nowadays accepted. As a matter of fact, there is a number of studies which show incoherent results with respect to this. To elucidate this question, protein targeting is mandatory since genomic detection is insufficient to conclude on functional expression.

For this purpose, we employed a widely used polyclonal TRPM6 antibody from an established manufacturer to investigate TRPM6 immunostaining in murine kidneys. In addition, hematoxylin and eosin and periodic acid-Schiff stains were performed. To validate indicated proximal and distal tubular TRPM6 expression, conditional *trpm6* knockout mice were used to test antibody specificity.

## Methods

### Animals

For immunohistochemistry, kidney samples were obtained from four 4–5-month old male physiological wild-type mice with C57BL/6 J genetic background with a breeding permit according to Sect. 11 for all animals and an annual disclosure statement for euthanized animals for scientific purpose according to Sect. 4 paragraph 3 of the German Animal Welfare Act. The breeding and keeping of these animals are not considered as an animal experiment and do not require an additional permit. Animals were sacrificed after phenobarbital application by bleeding. The organs were placed in 4% buffered formalin for 24 h. Afterwards, they were paraffin-embedded using a tissue embedder (Tissue-Tek VIP 6 Al, Sakura, Umkirch, Germany) with the following steps: water (1 h), 70% isopropanol (2 × 1 h), 90% isopropanol (1 × 1 h, 1 × 2 h), 100% isopropanol (2 × 1 h, 1 × 2 h), xylene (1 × 1 h, 1 × 2 h), and finally, liquid paraffine (4 × 1 h; Carl Roth, Karlsruhe, Germany). All methods were performed in accordance with the relevant guidelines and regulations.

For immunofluorescence, kidney samples were obtained from four 8-week-old mice, kept in 18% sucrose overnight, and embedded in tissue freezing medium, as previously described (Chubanov et al. 2016). Ten micrometer thick sections were cut and stored at − 80 °C. Genetics of the two Sox2cre-induced epiblast *trpm6* knockout mice and the two control *trpm6*^fl/+^ littermates have previously been described (Chubanov et al. 2016). These experiments were done in accordance with the European Union Animal Welfare Act and were approved by the local council on animal care (Permit No.: 55.2-1-54–2532-134-13 from the Government of Oberbayern, Germany).

### Hematoxylin and eosin histology

Hematoxylin and eosin (H&E) staining was performed as previously described (Englisch et al. 2024b; Kirstein et al. 2024a) using routine techniques (Cardiff et al. 2014).

### Periodic acid-Schiff histology

For periodic acid-Schiff (PAS) reaction, paraffin was removed, and slides were incubated in 1% periodic acid solution (Carl Roth, Darmstadt, Germany), followed by two rinses with distilled water. An incubation step with Schiff`s reagent (Carl Roth, Darmstadt, Germany) for 1 h at room temperature followed afterwards. The slides were washed three times for 5 min each in sulfur dioxide water, followed by incubation in running tap water for 15 min. After a short rinse in distilled water, the slides were stained using Ehrlich-hematoxylin (Carl Roth, Darmstadt, Germany) for 15 min. The counterstain was differentiated using HCl-alcohol for 30 s. Afterwards, the slides were rinsed twice in distilled water. For bluing, the slides were incubated in running tap water for 10 min. At the end, the slides were dehydrated in ascending alcohol series and three portions of xylene and covered with a coverslip using Histo-Kitt II mounting medium (Carl Roth, Darmstadt, Germany).

### Immunohistochemistry

For immunohistochemical staining, paraffin was removed, and antigen recovery was done using citrate buffer (60 min, 95 °C). The primary antibody (polyclonal TRPM6, lyophilized, ACC-046, 1:100, Alomone Labs, Jerusalem BioPark, Israel) was applied overnight and at room temperature. Rabbit serum was employed to prepare the negative control. A peroxidase-labeled secondary antibody (HRP anti-rabbit goat, A10547; Invitrogen AG, Carlsbad, CA, USA) and diaminobenzidine tetrahydrochloride (DAB) (SK-4103 Vector Laboratories, Burlingame CA, USA) were added, followed by counterstaining with hematoxylin (Carl Roth, Karlsruhe, Germany). The manufacturer (Alomone Labs, Jerusalem BioPark, Israel) indicated that specificity of the TRPM6 antibody was blocking-peptide-validated and designed to detect the corresponding channels in mouse, rat, and human tissue. Specifically, the peptide (CVKDYDLERGPDEK) was designed to detect the amino acid residues 802–815 (first extracellular loop) of mouse TRPM6.

### Immunofluorescence

Staining was performed as described previously (Yu et al. 2023). In short, sections were washed with phosphate-buffered saline (PBS) and blocked in 10% donkey serum/3% bovine serum albumin (BSA)/0.3% Triton X-100 in PBS for 1 h at room temperature. Sections were incubated with primary antiserum (polyclonal TRPM6, lyophilized, ACC-046, 1:250, Alomone Labs, Jerusalem BioPark, Israel) overnight at 4 °C followed by secondary antiserum (#711-175-152, 1:500, Jackson ImmunoResearch) at room temperature for 2 h. Cell nuclei were counterstained with bisbenzimide (Sigma-Aldrich, Saint-Louis, US).

### Visualization

Microphotographs from H&E, PAS, and immunohistochemistry were captured using Nano Zoomer S210 (Hamamatsu, Japan)-digitalized slides and the NDP.view2 Image viewing software (U12388-01). Microphotographs from immunofluorescence were captured using a ZEISS Axio Imager 2 microscope (Zeiss, Oberkochen, Germany) and analyzed with the ZenBlue Software (Zeiss, Oberkochen, Germany).

### Analysis

Semiquantitative analysis of immunostaining was performed as previously described (Diebolt et al. 2024). Briefly, five representative sections of each structure of interest (e.g., cortical proximal tubule) were defined as regions of interest (ROI). Thresholding was performed to detect DAB-positive areas within the ROIs. The threshold value was between the darkest brown staining (0) and the lightest still visible brown coloration (160). Areas with a value between 0 and 55 were classified as 3, between 56 and 110 as 2, and between 111 and 160 as 1. A weighted DAB area score between 0 and 3 normalized to the included sum of hematoxylin and DAB-positive area, which resembles a pixel-wise H-score was obtained (Ram et al. 2021).

Data are displayed as mean value ± standard deviation. The nonparametric Kruskal-Wallis test followed by Dunn’s multiple comparisons test was used to compare the ranks of several independent groups. *P* values (*P*) were two-tailed and significant when < 0.05. The GraphPad Prism Software Package (GraphPad, Version 10.3.0) was used for statistical analysis.

## Results

**Fig. 1:**
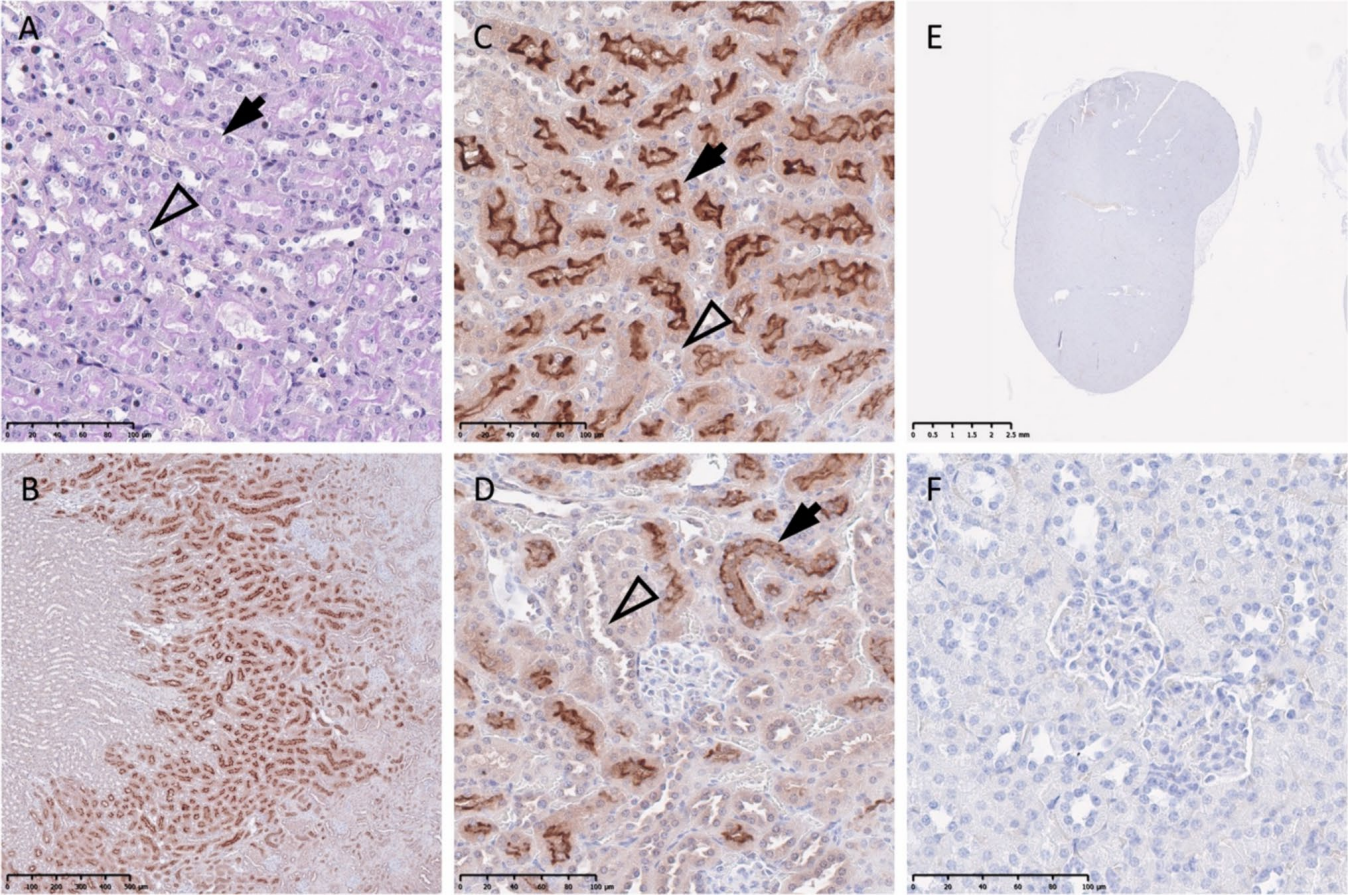
Periodic acid-Schiff staining (**A**), TRPM6 immunohistochemical staining (**B**, **C**, and **D**), and negative control staining for the TRPM6 immunohistochemistry (**E** and **F**) of mouse kidney tissue. Arrows indicate proximal tubules, and arrowheads distal tubules. Scale bars, 2.5 mm (E); 500 µm (B); 100 µm (A, C, D, and F)

**Fig. 2:**
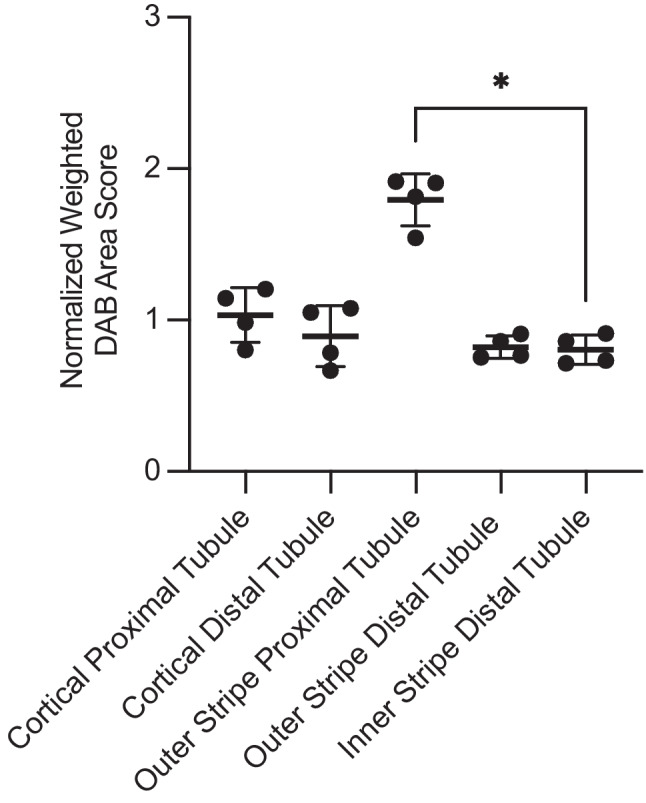
Normalized weighted diaminobenzidine (DAB) area score of TRPM6 immunostaining of mouse kidney tubules. Each point represents an individual mouse sample. The bars indicate the mean value with the standard deviation. The non-parametric Kruskal-Wallis and post-hoc Dunn’s multiple comparisons test (shown) were applied. Significant differences are indicated (*; *P* < 0.05)

H&E stains indicated well conserved tissue samples. PAS stains enabled secure identification of prominent brush borders in proximal tubules (Fig. 1A). Immunohistochemistry displayed a positive immunosignature in the mouse kidney (Fig. 1B). Proximal tubules within the outer stripe of the outer medulla were stained (mean value = 1.8 ± 0.2, Fig. 2), particularly their brush borders (Fig. 1C). Distal tubules appeared slightly stained within the outer stripe of the outer medulla (mean value = 0.8 ± 0.1, Figs. 1C and 2). Brush borders of cortical proximal tubules were particularly stained, whereas cortical proximal (mean value = 1.0 ± 0.2) and distal (mean value = 0.9 ± 0.2) tubules overall appeared slightly stained (Figs. 1D and 2). There was a significant difference between outer stripe proximal tubules and inner stripe distal tubules (mean value = 0.8 ± 0.1, *P* = 0.03, Dunn’s test, Fig. 2). Negative controls were free of DAB-specific coloration (e.g., cortical distal tubule = 0.015, Fig. 1E and F).

**Fig. 3:**
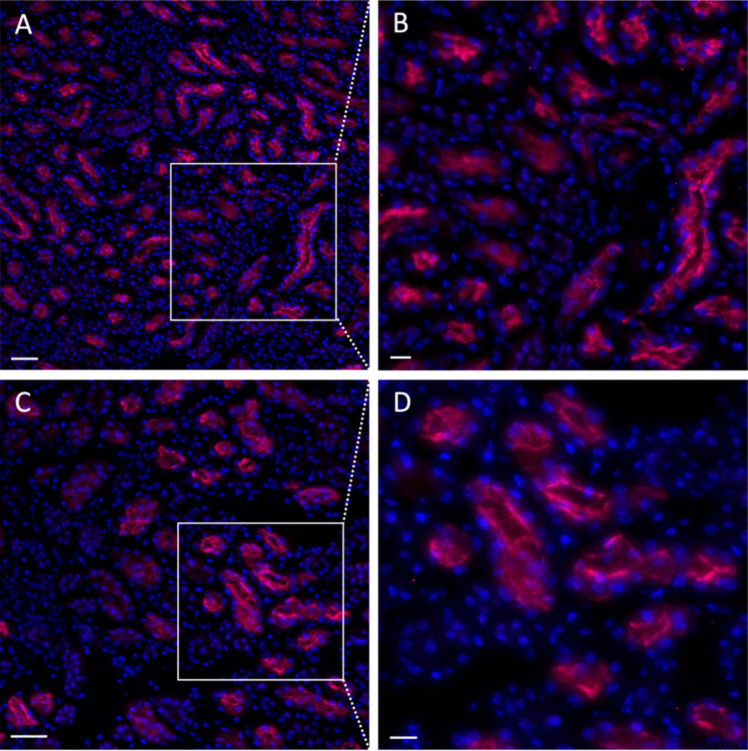
TRPM6 immunofluorescence staining of control wildtype mouse kidney tissue (**A** and **B**) and corresponding *trpm6* knockout tissue (**C** and **D**). Scale bars, 25 µm (A and C); 10 µm (B and D)

With regard to immunofluorescence, antibody incubation with wildtype kidney tissue displayed a tubular staining pattern (Fig. 3A and B). Incubation with the conditional knockout mouse tissue showed similar staining patterns (Fig. 3C and D).

## Discussion

### Trpm6 knockout and antibody specificity

The aim of this study was to investigate the distribution profile of TRPM6 in the mouse kidney using immunohistochemical methods with a regularly employed antibody. We detected a TRPM6 immunosignature in proximal and distal tubules, whereby the brush borders of the former were particularly stained. Immunofluorescence confirms this pattern by showing an accentuated staining at brush borders, which are mostly present in proximal tubules. As the literature suggests that TRPM6 is mainly expressed in the DCT, we were interested in whether our results could be trusted. We thus used a conditional knockout mouse and found that the staining pattern with the aforementioned antibody did not differ between wildtype and knockout tissue. The knockout was created by inducing a premature stop codon leading to the absence of the entire region containing the transmembrane domains of TRPM6. The possibility that the antibody recognizes parts of the yet expressed amino acids 1–676 was discussed, but according to the manufacturer, the antibody recognizes the amino acid residues 802–815 of the first transmembrane domain.

Of note, the *trpm6* knockout mouse strain was extensively characterized previously (Chubanov et al. 2016). The null mutation in *trpm6* which was induced by the deletion of exon 17 in the *trpm6* gene and led to the frameshift mutation, was verified using multiple approaches, including the control cross of + / − mice with an alternative null mutation in *trpm6* (Chubanov et al. 2016). The lack of TRPM6 protein in *trpm6* knockout tissues was confirmed by immunostaining of kidney tissue sections (Chubanov et al. 2016). The Western blot analysis of tissue extracts was problematic owing to a very low expression of TRPM6 protein and because only a minor fraction of the kidney cells expresses TRPM6. The impact of the frameshift mutation on *trpm6* mRNA was demonstrated by in situ hybridization (Chubanov et al. 2016).

Of note, the same antibody as used here was employed several times to investigate TRPM6 in the kidney (Lee et al. 2016; Meurer and Hocherl 2019; Ng et al. 2021; Nie et al. 2024). Antibody specificity had also been investigated using corresponding blocking peptides and had been labeled as good (Andriule et al. 2022). This shows that TRPM6 is difficult to detect with high specificity and that blocking peptide validation does not reach the level of quality of knockout validation.

It remains unclear what antigen the antibody from this manufacturer recognized herein. Cross binding with TRPM7 would have been conceivable. Indeed, next to tremendous analogies, functional heteromeric assembly of TRPM6 with TRPM7 has been demonstrated (Chubanov et al. 2004). However, TRPM7 is ubiquitously expressed and would presumably not be restricted to the proximal tubule’s brush border. Instead, a component of the brush border would be a more promising candidate responsible for unspecific binding.

### Literature review

The first reports on renal TRPM6 research date back to the early 2000’s, when Schlingmann et al. detected *trpm6* gene mutations leading to autosomal-recessive HSH (Schlingmann et al. 2002). Segmental distribution analysis of *trpm6* cDNA in microdissected rat nephrons yielded strong signals in the DCT and weaker signals in the proximal convoluted tubule and collecting duct (Schlingmann et al. 2002). *trpm6* mRNA was also detected in single human distal renal tubule cells using in situ hybridization (Schlingmann et al. 2002). At the same time, Walder et al. supported these findings by revealing abundant *trpm6* mRNA expression in human and mouse kidneys (Walder et al. 2002). Later, Voets et al. investigated the cellular and subcellular localization of the *trpm6*-encoded TRPM6 protein in C57BL/6 mouse tissue (Voets et al. 2004). In contrast to the genomic analysis from Schlingmann et al. (Schlingmann et al. 2002), the TRPM6 protein was restricted to the apical side of DCT cells (Voets et al. 2004). Voets et al. therefore promptly discussed the potential methodical weakness of tubular microdissection as prone to impurities and therefore potentially causative of the non-distal convoluted tubule polymerase chain reaction (PCR)-signals observed by Schlingmann et al. (Schlingmann et al. 2002) (Voets et al. 2004). Another possible explanation for the observed discrepancies is the investigated species, that was different between the two studies. As pointed out by Loffing and Kaissling in the case of renal sodium and calcium transporters, protein distribution among species often varies, although differences between rats and mice have been small, in contrary to differences between rodents and humans (Loffing and Kaissling 2003). A likely conclusion might therefore also be that *trpm6* is expressed in rat proximal tubules and collecting ducts, as suggested by Schlingmann et al. (Schlingmann et al. 2002). In the literature, the aforementioned studies from Schlingmann et al. (Schlingmann et al. 2002), Walder et al. (Walder et al. 2002), and Voets et al*.* (Voets et al. 2004) are the most important primarily investigating *trpm6* expression- and/or TRPM6 protein-localization in rodent and/or human kidneys. Many following publications focused on experimental topics and introduced a DCT-restricted TRPM6 localization referencing to one or several of the previously enumerated studies (Schlingmann et al. 2002; Walder et al. 2002; Voets et al. 2004). Interestingly, however, while investigating the effect of dapagliflozin and magnesium supplementation on renal magnesium handling factors—including the TRPM6 channel—in the context of metabolic syndrome—here mimicked by high-fructose diets, Ng et al. also performed TRPM6 immunohistochemistry of male Sprague-Dawley rat sections (Ng et al. 2021). Although apparently not entirely commented in the publication (Ng et al. 2021), the presented sections featured a strong TRPM6 immunosignal in distal tubules but also weaker signals in proximal tubules and in selected glomerular cells—with collecting ducts being not displayed (Ng et al. 2021). Furthermore, fructose, fructose + dapagliflozin, and fructose + magnesium diets/treatments seemingly increased the TRPM6 protein expression in all localizations, which supports the involvement of TRPM6 in both physiological and pathophysiological states including metabolic syndrome, that may potentially not be restricted to DCTs (Ng et al. 2021).

Jiang et al., for instance, investigated the predictive value of serum magnesium levels in Gitelman syndrome patients—an autosomal recessive renal salt-wasting condition—with respect to disease’s severity (Jiang et al. 2014). In this context, Gitelman syndrome kidney sections and control sections of hematuria-patient kidneys with minor glomerular lesions were allocated to TRPM6 staining (Jiang et al. 2014). The presented immunohistochemical sections did not allow statements restricting the immunoreactive signal to DCTs (Jiang et al. 2014). Neither did immunofluorescence, as TRPM6-labeling was not only detected in colocalization with the sodium-chloride co-transporter (NCC) (Jiang et al. 2014). Both points were apparently not mentioned or discussed by the authors, probably due to the differing focus of this publication. In trans-species analogy, while investigating renal adaptative mechanisms to gentamycin-induced mineral loss, Lee et al. submitted Sprague-Dawley rat sections to immunofluorescence and localized the TRPM6 protein to the apical membrane of both Henle’s loop—composed of straight segments of the proximal and distal tubules at the edges and intermediate tubule segments in the middle (Reilly and Ellison 2000)—and the DCT (Lee et al. 2012).

## Conclusion

The herein employed commercially available antibody did not detect a proven signal of TRPM6 in our hands. However, this antibody was employed in several studies investigating renal disorders. A literature review showed that there are hints of lack of specificity or extended TRPM6 expression beyond the DCC. This report is thus critical since outlining the trickiness of versatile targets, such as TRPM6.

Future research will have to address the question, as to whether TRPM6 expression is restricted to the DCC and how the expression differs between species including humans, which can impact the understanding and yield eventual novel treatment targets of associated disorders in humans.

## Data Availability

All source data for this work (or generated in this study) are available upon reasonable request.
